# The relationship between self-regulation, cognitive flexibility, and resilience among students: a structural equation modeling

**DOI:** 10.1186/s40359-024-01843-1

**Published:** 2024-06-07

**Authors:** Mohammad Nakhostin-Khayyat, Mahmoud Borjali, Maryam Zeinali, Deniz Fardi, Ali Montazeri

**Affiliations:** 1 Department of Clinical Psychology, Islamic Azad University, Electronic Campus, Tehran, Iran; 2 Department of Psychology, Kharazmi University, Karaj, Iran; 3 South Tehran Branch, Payame Noor University, Tehran, Iran; 4 Independent Registered Psychotherapist, Tehran, Iran; 5 Health Metrics Research Center, Iranian Institute for Health Sciences Research, ACECR, Tehran, Iran; 6 Faculty of Humanity Sciences, University of Science and Culture, Tehran, Iran

**Keywords:** Cognitive enhancers, Iran, Self-regulation, Students, Resilience

## Abstract

**Background:**

Cognitive flexibility is an important construct that contributes to one’s own thoughts, behaviors, and feelings to achieve his or her goals. Thus, it could play an essential role in students’ educational achievements. This study aimed to investigate the mediating role of cognitive flexibility in the relationship between self-regulation and resilience among students.

**Method:**

This was a cross-sectional study conducted on a sample of students during the 2022 and 2023 academic years. Students were selected from Tehran and Karaj universities (two metropolitans in central Iran). Data collection instruments included the Bouffard’s Self-Regulation Scale, the Cognitive Flexibility Inventory (CFI), and the Connor-Davidson Resilience Scale (CD-RSC). Subsequently, the data were analyzed using structural equation modeling via SPSS and AMOS software to examine the relationships among variables.

**Results:**

In all 302 students participated in the study. The mean age of students was 25.8 (SD = 4.05) years. The findings indicated that self-regulation had a marked positive direct effect on cognitive flexibility (β = 0.23, *p* < 0.001), and resilience (β = 0.88, t = 19.50, *p* < 0.001). Similarly, cognitive flexibility displayed a strong positive influence on resilience (β = 0.1, *p* < 0.001) it showed an indirect mediating role between self-regulation and resilience (0.02), while resilience demonstrated a negative indirect effect on self-regulation and cognitive flexibility (-0.23). The goodness of fit indices validated the proposed model. Furthermore, the analysis revealed the significance of the final model’s direct path coefficients, underscoring the mediating role of cognitive flexibility between self-regulation and resilience among students.

**Conclusion:**

The findings indicated a pivotal interrelationship among self-regulation, cognitive flexibility, and resilience in students. The significant positive relationship among these constructs underscores the importance of fostering cognitive flexibility practices and self-regulation in educational settings.

## Introduction

The transition into university marks a pivotal and challenging period in an individual’s academic journey. As students embark on this new phase, they not only shoulder crucial roles and responsibilities toward future contributions to public health but also grapple with numerous pressures and changes [1]. These challenges have the potential to impact students’ mental health profoundly. For instance, 60% of university students reported high stress levels during their academic years [2, 3]. It is thus evident that any attempts to improve mental health among students are of prime importance as such it has been shown that cognitive flexibility is an essential factor for improving mental health. However, the relationship between cognitive flexibility and improved mental health depends on several other intervening variables including self-regulation and resilience. To improve mental health and academic achievements among students one needs to improve self-regulation and resilience among this population which in turn could improve cognitive flexibility to improve mental health and successful educational attainment ultimately. In the following sections, we briefly explain these relationships using the current evidence on the topic [4, 5].

## Self-regulation

The ability to self-regulate is highly beneficial for both individual well-being and societal functioning, influencing diverse areas such as health, lifespan, criminal behavior, financial habits, job performance, and relationship contentment. Self-regulation stands as a fundamental element of human functioning, playing a pivotal role in enabling the effective pursuit and achievement of individual objectives [6].

self-regulation in education refers to the ability of students to regulate their own learning process, including their cognitive, motivational, and behavioral dimensions of academics. Research indicates that students who can self-regulate are more successful as learners [7].

A significant individual determinant of student success or failure is self-efficacy, which is greatly influenced by various factors, among which self-regulation strategies stand out as particularly crucial [8]. Self-regulation, a foundational facet of human performance, plays a pivotal role in the pursuit and attainment of personal goals. Self-regulation constitutes a behavioral, cognitive, emotional, and physiological framework encompassing individuals’ conscious or unconscious efforts to regulate states or responses. Ashmita and Analakshmi’s study on the relationship between self-regulation and attachment style with resilience and academic progress among at-risk rural adolescents revealed interesting findings. The results demonstrated that self-regulation was the sole predictor of resilience. Moreover, it was found that self-regulation positively predicted academic achievement [9]. Similarly, a study reported that students with stronger self-regulation skills generally demonstrate greater overall success both academically and socially [10].

Research held by Martini Jamaris and Sofiah Hartati demonstrated that undergraduate students can manage their academic self-regulation. The ability is reflected in (1) planning their study goal, (2) managing their behavior to achieve their study goal, and (3) the academic achievements of the undergraduate students, in which, they achieve their study goal well. The research result was the same as the results of the research on self-regulation of graduate students and its impact on their academic achievements [11].

## Cognitive flexibility

Cognitive flexibility in an important psychological construct that have been studied in various contexts. Research has shown that cognitive flexibility, which refers to the ability to adapt to new information and changing circumstances, is related to self-regulation, which involves managing one’s thoughts, emotions, and behaviors to achieve goals [12]. Cognitive flexibility refers to the mental ability to adapt and switch between different cognitive tasks or perspectives. In the context of educational achievement, cognitive flexibility plays a crucial role in a student’s capacity to navigate diverse learning situations, grasp new concepts, and solve complex problems. Individuals with higher cognitive flexibility tend to exhibit enhanced adaptability, creativity, and resilience when faced with academic challenges. This cognitive skill allows students to approach learning with an open mind, explore alternative strategies, and adjust their thinking in response to varying academic demands. Ultimately, cognitive flexibility contributes to more effective learning experiences and improved educational outcomes [13]. A study reported that students possessing suitable cognitive flexibility have the capacity to appraise various situations from multiple viewpoints. They can deeply analyze scenarios, assess different alternatives, and select fitting strategies to navigate unfamiliar challenges and circumstances [14]. In a different study conducted by Korhan et al., it was demonstrated that individuals with effective self-regulation and high cognitive flexibility experienced lower levels of test anxiety compared to those with low cognitive flexibility and ineffective self-regulation [15].

Cognitive flexibility is an effective cognitive skill for self-regulation. Research held by İsmail Ay showed that cognitive flexibility and mindfulness are significant predictors of self-regulation. Accordingly, cognitive flexibility predicted 20% of the variance in self-regulation, while mindfulness predicted 11% of the variance. Furthermore, the results indicated that together, these two variables explain a substantial portion (46%) of the variance in self-regulation [16].

## Resilience

Resilience is the process of effectively negotiating, adapting to, or managing significant sources of stress or trauma. It involves the capacity for adaptation and ‘bouncing back’ in the face of adversity, which is facilitated by assets and resources within the individual, their life, and environment [17]. Resilience can be characterized as an individual’s capacity to adapt constructively to stressful and challenging circumstances. Resilience emerges when individuals confront threatening and challenging situations head-on, rather than evading them. Furthermore, a significant correlation exists between resilience and overall life satisfaction among students [18].

Consequently, students who possess a high degree of academic resilience demonstrate greater tolerance in stressful situations, exhibit enhanced cognitive flexibility when faced with stressors, and despite challenging circumstances, persistently strive to attain their objectives [19]. In a study conducted by Burton et al., cognitive flexibility was identified as one of the five critical and influential factors contributing to resilience. Individuals with higher resilience tend to perceive negative situations more realistically and flexibly compared to those with relatively lower resilience [20]. Research conducted by Artuch-Garde et al. showed The ability to self-regulate behavior is one of the most important protective factors with resilience and should be fostered especially in at-risk youth. Relationships between them were significant and positive. Learning from mistakes (self-regulation) was a significant predictor of coping and confidence, tenacity and adaptation, and tolerance to negative situations (resilience). Likewise, low-medium-high levels of self-regulation correlated with scores on resilience factors [21].

## The study hypotheses

We hypothesized that the higher levels of cognitive flexibility in students will be positively correlated with increased self-regulation and resilience. Specifically, we predict that students with stronger cognitive flexibility will demonstrate greater self-regulation and resilience compared to those with lower levels of congnitive flexibility.

## Research question

The studies conducted showed that self-regulation, cognitive flexibility, and resilience generally exhibited a positive and significant correlation with managing and maintaining psychological stability. However, given the scarcity of research specifically addressing the role of self-regulation, cognitive flexibility, and resilience among students, and considering that most studies have concentrated on the implications of these constructs in other fields and diverse societies, this study sought to concentrate specifically to explore the mediating role of cognitive flexibility in the relationship between self-regulation and resilience in students.

## Methods

### Design and participants

This was a cross-sectional study carried out on samples of university students assessing the relationship between self-regulation, cognitive flexibility, and resilience. A total of 302 students participated in the study (146 men and 156 women). The mean age of students was 25.8 (SD = 4.05) years ranging from 18 to 35. Of these, 32.1% of the participants were undergraduate, the remaining students were postgraduate students (55.3% master, and 12.6% Ph.D. students). The characteristics of students are shown in Table 1.

**Table 1:** Distribution of Samples (*n* = 302)

Gender	Frequency	Percentage
Female	156	51.7
Male	146	48.3
**Educational level**		
Bachelor	97	32.1
Masters	167	55.3
Doctorate	38	12.6
**Age group (year)**		
18 to 20	28	9.27
21 to 25	125	41.39
26 to 30s	107	35.43
31 to 35	42	13.91

### Sampling and sample size

The study employed a convenient sampling method. The statistical population of the study included all university students from two metropolitans (Tehran, and Karaj), Iran during the academic year 2022–2023. To estimate the sample size, we followed the recommendation by Hair et al., which suggested a minimum of 200 individuals for conducting a structural equation modeling [22]. Due to time constrain and difficulty in traveling to collect data from several universities, we decided to collect data online. As such we invited the students via Telegram application targeting students’ groups. The message included a link to an Iranian platform (Porsline) where the students could sign the consent form and access the study questionnaires. The inclusion criteria consisted of the following conditions: (1) signing a written informed consent form, (2) being a student in the current semester of 2022–2023, and (3) aged 18 to 35 years.

### Data collection

Participants were initially briefed about the study’s objectives. They retained the option to withdraw from the study at any point. The entire study, including data collection, adhered to the ethical standards established by our research committee. No financial incentives were offered to the participants for their involvement. They then proceeded to complete the online questionnaires. The study measures are described in the following section.

### Measures

In addition to a demographic questionnaire collecting information on participants’ age, gender, and education, the following questionnaires were administered:

**Connor–Davidson Resilience Scale (CD-RISC)**: The CD-RISC is a 25-item questionnaire that assesses the individual’s ability to cope with stress and adversity. Items are rated on a 5-point Likert scale ranging from 0 (not true at all) to 4 (‘true nearly all the time). According to exploratory factor analysis, the CD-RISC is a multidimensional instrument measuring five factors as follows: personal competence/tenacity, positive acceptance of change/secure relationships, trust in one’s instincts/tolerance of negative affect, spirituality, and control. The Preliminary research on the CD-RISC’s psychometric properties in the general population and clinical samples revealed sufficient internal consistency, convergent and divergent validity, and test-retest reliability [23]. psychometric properties of the Iranian version of CD-RISC are well documented. As such the internal consistency of the questionnaire as measured by Cronbach’s alpha was reported to be 0.89 [24]. The current study also obtained an alpha value of 0.91, which is well above the acceptable threshold.

**Cognitive Flexibility Inventory (CFI)**: The CFI is a 20-item self-report questionnaire developed for aspects of cognitive flexibility that enable people to challenge and replace maladaptive thoughts with more adaptive ones. Items are rated on a 7-point Likert-type scale to define the respondent’s approach to challenging situations accurately. The CFI assesses three factors as follows: Alternatives, Control, and Alternatives to human behavior [25]. . Dennis and Vander Wall reported that CFI had good to excellent internal consistency, and test-retest reliability was high for the CFI and its subscales. The Iranian version of the CFI also showed desirable reliability and validity. The results obtained from factor analysis indicated three factors (Control, Alternatives, and Alternatives for Human Behaviors) that jointly explained 56.02% of the variance observed. The test-retest and Cronbach’s alpha coefficients for the Iranian version of CFI were 0.71 and 0.90, respectively [26]. In this study, the alpha coefficient for the CFI was 0.90.

**Buford’s Self-Regulation Questionnaire**: The 14-item self-regulation questionnaire was developed by Buford et al. was validated in Iran among a sample of university students standardized by Kadivar [27, 28]. The reliability coefficient of the questionnaire based on Cronbach’s alpha was calculated to be 0.71. The validities of the sub-scales of cognitive and metacognitive strategies were 0.70 and 0.68, respectively. Regarding the structure, the factor results showed that the correlation coefficient of the questions was acceptable, and the evaluation tool consisted of two factors. The value of the factors was acceptable, and the tool could determine 0.52 of the self-report variances. The structural validity was satisfactory. There were five possible answers for each question: “I totally agree,” “I agree,” “I’m not sure,” “I disagree,” and “I totally disagree.” Each question was scored from 1 to 5, except for questions 5, 13, and 14, which were scored in the reverse [28, 29].

### Statistical analysis

Descriptive statistics were used to explore the data. To achieve the study objective we first assessed the correlation among self-regulation, cognitive flexibility and resilience. Then to examine the association between self-regulation and resilience with mediating variable (cognitive flexibility) structural equation modeling (SEM) was performed. In fact, we were interested to see to what extent cognitive flexibility could explain variance in self-regulation and resilience. The analysis served to assess the degree of alignment between the theoretical-causal model and the empirical data. The data were analysed using SPSS-27 and AMOS software.

## Results

### Distribution of research variables

To ascertain the nature of data distribution, the Kolmogorov-Smirnov test, skewness, and kurtosis techniques were implemented. The outcomes of these assessments can be found in Table 2. The findings outlined that the p-value resulting from the Kolmogorov-Smirnov test for the variables exceeded the threshold of 0.05. This suggests that the distributions of resilience, flexibility, self-regulation, and their respective components did not significantly deviate from a normal distribution. Thus be inferred that the data distribution aligns closely with normality.

**Table 2:** Distribution of research variables

	Kolmogorov-Smirnov^a^	Mean	Std. Deviation	Skewness	Kurtosis
**Self-regulation**					
10. Cognitive	0.144	3.4655	0.81355	− 0.876	0.242
9. Metacognitive	0.147	3.4172	0.97100	-1.131	0.871
**Cognitive flexibility**					
8. Replacing ineffective thoughts with effective ones	0.169	4.9331	1.38404	− 0.620	− 0.784
7. Control	0.104	3.5337	0.76602	0.070	− 0.307
6. Alternatives for human behaviors	0.128	4.7533	1.50069	− 0.217	-1.090
**Resilience**					
5. Perception of individual competence	0.195	2.3891	1.00549	-1.053	0.434
4. Trusting individual instincts and tolerating negative emotions	0.132	2.2502	0.63688	− 0.537	0.227
3. Positive acceptance of change and secure relationships	0.220	2.3742	1.02979	-1.109	0.199
2. Control	0.180	2.3389	0.97607	-1.028	0.813
1. Spiritual influences	0.187	2.3096	1.22854	− 0.267	-1.069

### Correlation among self-regulation, cognitive flexibility, and resilience

To examine correlation among the components of self-regulation, cognitive flexibility, and resilience, Pearson’s moment correlation coefficient was employed. The outcomes are detailed in Table 3. The results revealed a significant positive correlation between the components of self-regulation, cognitive flexibility, and resilience. Given a meaningful relationship among variables, the mediating role of cognitive flexibility in the relationship between self-regulation and resilience was explored (See Table 4).

**Table 3:** Pearson correlation among constructs of the study questionnaires

	10	9	8	7	6	5	4	3	2	1
**Self-regulation**										
10. Cognitive	1									
9. Metacognitive	0.818^**^	1								
**Cognitive flexibility**										
8. Replacing ineffective thoughts with effective ones	0.277^**^	0.221^**^	1							
7. Control	0.198^**^	0.173^**^	0.497^**^	1						
6. Alternatives for human behaviors	0.192^**^	0.129^*^	0.801^**^	0.301^**^	1					
**Resilience**										
5. Perception of individual competence	0.757^**^	0.808^**^	0.251^**^	0.292^**^	0.109	1				
4. Trusting individual instincts and tolerating negative emotions	0.635^**^	0.719^**^	0.242^**^	0.344^**^	0.144^*^	0.816^**^	1			
3. Positive acceptance of change and secure relationships	0.742^**^	0.755^**^	0.252^**^	0.252^**^	0.124^*^	0.877^**^	0.689^**^	1		
2. Control	0.707^**^	0.753^**^	0.231^**^	0.298^**^	0.142^*^	0.872^**^	0.825^**^	0.811^**^	1	
1. Spiritual influences	0.507^**^	0.654^**^	0.150^**^	0.060	0.087	0.647^**^	0.555^**^	0.695^**^	0.555^**^	1

*Correlation is significant at the 0.05 level (2-tailed)

**Correlation is significant at the 0.01 level (2-tailed)

**Table 4:** Total, direct, and indirect standard coefficients in the model

Paths	Total effect	Direct effect	Indirect effect
Self-regulation over cognitive flexibility	0.23	0.46	− 0.23
Self-regulation over resilience	0.88	0.86	0.02
Cognitive flexibility over resilience	0.10	0.10	− 0.00
Resilience over cognitive-flexibility	− 0.25	− 0.26	0.00

### Summary of model findings

**Fig. 1:**
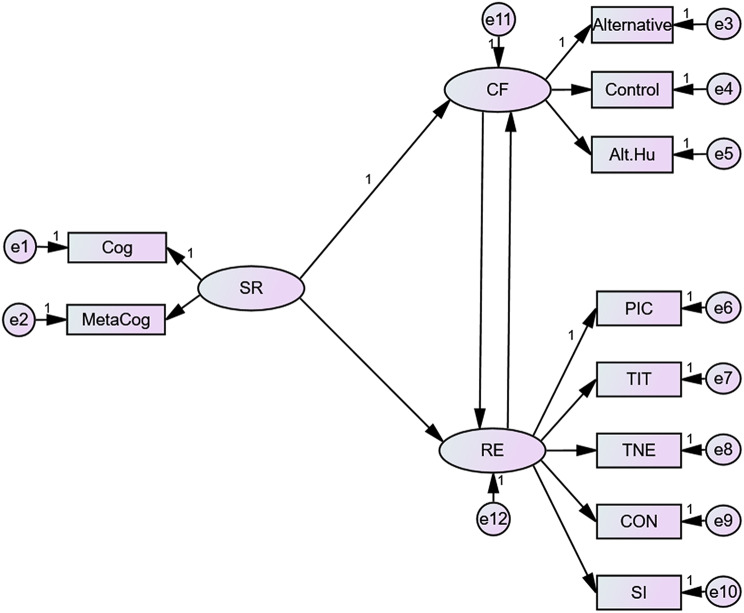
The relationship between self-regulation (SR), cognitive flexibility (CF), and resilience (RE) derived from the structural equation modeling

Utilizing the structural equation modeling (SEM), as illustrated in Fig. 1, we examined the relationships between self-regulation, cognitive flexibility, and resilience. The findings indicated that self-regulation had a marked positive direct effect on cognitive flexibility (β = 0.23, *p* < 0.001), and resilience (β = 0.88, t = 19.50, *p* < 0.001). Similarly, cognitive flexibility displayed a strong positive influence on resilience (β = 0.1, *p* < 0.001) it showed an indirect mediating role between self-regulation and resilience (0.02), while resilience demonstrated a negative indirect effect between self-regulation and cognitive flexibility (-0.23). Assessing the model’s fit using established indices, such as the chi-square to degrees of freedom ratio and the Comparative Fit Index (CFI), yielded acceptable thresholds, verifying the model’s appropriateness. The model’s pathway details can be found in Table 5. Overall, the results compellingly highlight cognitive flexibility’s mediating role in the dynamic between self-regulation and resilience among students. For further information, the model fit indices str presented in Table 6.

**Table 5:** Summary of model findings

Main hypothesis of the research			Standardized estimate	t-value	Significance
Self-regulation	→	Cognitive flexibility	0.46	-	0.001
Self-regulation	→	Resilience	0.86	19.50	0.001
Cognitive flexibility	→	Resilience	0.10	2.94	0.001
Resilience	→	Cognitive flexibility	− 0.26	-4.33	0.001

**Table 6:** Model fit indices

Model	Default model
CMIN/DF	2.068
RMR	0.030
GFI	0.966
AGFI	0.929
TLI (rho2)	0.982
CFI	0.990
RMSEA	0.060
HOELTER (0.05)	218
HOELTER (0.01)	256

## Discussion

The study investigated the objective of exploring the mediating role of cognitive flexibility in the relationship between self-regulation and resilience among students. Our findings corroborated that cognitive flexibility serves as a mediator between self-regulation and resilience. A strong and substantial positive direct effect was observed from Self-regulation to cognitive flexibility, and from cognitive flexibility to resilience, and a strong indirect effect from self-regulation to resilience with the mediating role of cognitive flexibility has been noted. Moreover, the direct impact of self-regulation on resilience in students was also noted to be negligible and the indirect impact of self regulation on cognitive flexibility was negative. The conceptual model displayed a suitable fit thereby substantiating the research hypothesis.

The results from the study further reinforce the findings from previous research, providing additional support for the crucial role of cognitive flexibility in fostering self-regulation and resilience in students. These results align well with prior studies that have elucidated the interconnected nature of these constructs. A rich body of evidence already underlines the significant positive associations among cognitive flexibility, self-regulation, and resilience, this attests to the replicability of the phenomena observed and the robustness of these constructs’ relations [30].

The study has substantiated the mediating role of cognitive flexibility, a concept that previous research has suggested but has been less conclusive. Our study thus fills a crucial gap in the literature by statistically confirming this mediator role, which will undoubtedly enrich the existing knowledge base and provide a platform for future research in this area.

The role of cognitive flexibility in influencing self-regulation and resilience among students can be elucidated as follows: students exhibiting a higher degree of cognitive flexibility tend to demonstrate enhanced self-regulation and resilience. This correlation indicates that individuals proficient in managing their thoughts, emotions, and behaviors, when confronted with academic or life challenges, are likely to exhibit adaptable thinking and flexible problem-solving strategies [31].

Such individuals are often better prepared to navigate their academic responsibilities, engage in meaningful social interactions, tackle complex problems, and deal effectively with the multifaceted demands of collegiate life. Further, those who display high cognitive flexibility are typically adept at setting realistic goals and formulating strategic plans to realize them. They possess the capacity to shift their cognitive strategies, adopt diverse approaches, and entertain various perspectives - crucial facets of self-regulation [32].

This bidirectional relationship illustrates that bolstering one of these characteristics can trigger the enhancement of the other. For instance, individuals with robust self-regulation skills may demonstrate greater adaptability and flexibility when faced with adversity, allowing them to engage with and navigate these challenges with greater ease. Conversely, individuals who exhibit elevated cognitive flexibility typically demonstrate superior capacity in formulating appropriate goals and devising effective strategies to attain them, This is indicative of the interdependence and mutually reinforcing relationship between cognitive flexibility and self-regulation.

### Limitations

While our findings offer promise, they should be interpreted considering certain limitations. First, our sample size was relatively small, which may compromise the generalizability of our results to broader populations. Second, as with many types of research, it was challenging to control for all potential intervening or disruptive variables that could influence our outcomes. Lastly, our study predominantly focused on participants within the age range of 18–35. This specificity limits the direct applicability of our findings to other age groups.

### Future directions

We recommend that future research on this topic should incorporate larger and more diverse samples to ensure broader applicability of the findings. Moreover, the exploration of this model across different educational levels within the student population could further enrich our understanding of these relationships. In practical terms, these findings carry significant implications for the educational sector. As such, it would be beneficial to investigate the efficacy of cognitive treatments and exercises geared toward bolstering self-regulation, with the aim of enhancing cognitive flexibility, resilience, and, ultimately, academic performance and mental well-being in students.

## Conclusion

In conclusion, the study revealed the central role of cognitive flexibility in mediating the relationship between self-regulation and resilience among students. Our findings not only resonate with previous research but also fill an existing gap by quantifying this relationship. The intertwined nature of these traits suggests that strengthening one could potentially enhance the others, emphasizing the need for an integrated educational approach. Given the implications for fostering adaptability and success in students, educators and policymakers should prioritize initiatives that emphasize these critical skills. This study sets a foundation for both future research and the development of targeted educational strategies.

## Data Availability

The data is available from the first investigator (MNK) on reasonable request.
